# “You Should Have Seen the Look on Your Face…”: Self-awareness of Facial Expressions

**DOI:** 10.3389/fpsyg.2017.00832

**Published:** 2017-05-30

**Authors:** Fangbing Qu, Wen-Jing Yan, Yu-Hsin Chen, Kaiyun Li, Hui Zhang, Xiaolan Fu

**Affiliations:** ^1^College of Preschool Education, Capital Normal UniversityBeijing, China; ^2^State Key Laboratory of Brain and Cognitive Science, Institute of Psychology, Chinese Academy of SciencesBeijing, China; ^3^Department of Psychology, University of Chinese Academy of SciencesBeijing, China; ^4^Institute of Psychology and Behavioral Sciences, Wenzhou UniversityWenzhou, China; ^5^School of Education and Psychology, University of JinanJinan, China; ^6^Department of Biostatistics, St. Jude Children’s Research Hospital, MemphisTN, United States

**Keywords:** self-awareness, facial expression, awareness rate, duration, intensity

## Abstract

The awareness of facial expressions allows one to better understand, predict, and regulate his/her states to adapt to different social situations. The present research investigated individuals’ awareness of their own facial expressions and the influence of the duration and intensity of expressions in two self-reference modalities, a real-time condition and a video-review condition. The participants were instructed to respond as soon as they became aware of any facial movements. The results revealed that awareness rates were 57.79% in the real-time condition and 75.92% in the video-review condition. The awareness rate was influenced by the intensity and (or) the duration. The intensity thresholds for individuals to become aware of their own facial expressions were calculated using logistic regression models. The results of Generalized Estimating Equations (GEE) revealed that video-review awareness was a significant predictor of real-time awareness. These findings extend understandings of human facial expression self-awareness in two modalities.

## Introduction

At some point in our lives, we are usually confronted with a situation in which someone says, “You should have seen the look on your face….” One typically attempts to recall one’s facial expression and ponders, “What was the look on my face?” to assess whether the facial expression expressed was appropriate in accordance with social norms, such as the feeling rules (Hochschild, 1979) and display rules (Ekman and Friesen, 1971). An accurate interpretation of one’s facial expression is important in every interpersonal interaction because a considerable amount of information about one’s affective state, status, attitude, cooperativeness, and competitiveness in social interactive situations is expressed and communicated to others through facial expressions (Ekman and Friesen, 1971; DePaulo, 1992; North et al., 2010, 2012). The misappraisal of facial expressions that we display to other people may have important consequences and may influence the course of the interaction. To prevent and mitigate the chances of misinterpreting our facial expressions, we need to possess a certain amount of emotional self-awareness, that is, what is expressed in our daily interactions with others (Hess et al., 2004).

Psychologists generally agree that individuals are experts at monitoring and perceiving their own emotional states and are capable of providing more accurate self-reports of their subjective experience of emotions and bodily experience than most other individuals could (Barrett et al., 2007; De Vignemont, 2014). Ansfield et al. (1995) noted an interesting dilemma in which we are rarely able to observe our own facial expressions, although others can see them. Hence, we often hear people say, “You should have seen the look on your face….” On many occasions, previous studies have noted discrepancies in humans’ subjective experience of their facial expressions. Riggio et al. (1985) assessed individuals’ self-perceived emotion-sending abilities by asking participants to express six basic emotions and rate their perceived success during the emotion-sending task. They observed that participants’ self-perceived emotion-sending ability was not significantly correlated with their actual emotion-sending ability. Barr and Kleck (1995) first videotaped participants’ facial expressions and asked them to rate their own facial expressiveness. Then, participants were shown the videotapes of their facial expressions. In their study, participants expressed surprise toward the inexpressiveness of their faces. This study inferred that people have stronger awareness of sensorimotor feedback but have weak facial display. These observations about humans’ subjective experiences of facial expressions raise an interesting question that warrants further investigation regarding the extent of individuals’ awareness of their own facial expressions. The literature offers very few studies in which researchers have directly investigated individuals’ awareness of their own facial expressions, including real-time awareness (referring to participants’ immediate self-reports of the occurrence of their facial expressions) and video-review awareness (referring to the extent to which participants can identify any facial movements in their face recordings).

Based on previous literature on human emotion, a crude conception of individuals’ awareness of facial expressions can be proposed. Specifically, sensory feedback of sufficient strength is required for individuals to become aware of their facial expressions. Support for this notion is provided by a statement posited by Ekman: “While there are sensations in the face that could provide information about when muscles are tensing and moving, my research has shown that most people don’t make much use of this information. Few are aware of the expressions emerging on their face until the expressions are extreme” (Ekman, 2009). Tomkins’s (1962) facial feedback hypothesis posited that the responses of the affected motor and glandular targets (the face, primarily) supply sensory feedback to the brain, which is subjectively experienced as emotion if it reaches consciousness. It can be inferred from this passage that an individual may only become aware of his or her facial expression and emotion if and only if sensory feedback from the facial muscles is strong enough to reach consciousness. These findings and other studies further suggest that awareness of facial expressions may be influenced by factors such as facial expression intensity, duration, and frequency (Ekman et al., 1980; Adelmann and Zajonc, 1989). Although direct evidence in support of this claim is scarce at this time, numerous studies investigating facial expressions and their recognition may provide indirect evidence in support of this notion. In previous facial expression recognition studies, manipulation of facial expression duration was observed to influence individuals’ recognition performance; specifically, the recognition rate decreased as a function of expression duration (Shen et al., 2012). Studies have also manipulated the intensity of facial expressions and observed that facial expressions with a higher intensity are recognized at a higher rate (Herba et al., 2006). Heuer et al. (2010) also investigated the detection and interpretation of emotional facial expressions by employing facial morphing paradigm and experimentally manipulated viewing conditions for emotion processing. Their results suggested as the facial expression intensity developing slowly, non-anxious controls and socially anxious individuals show different capacity of emotion onset perception, decoding accuracy, and interpretation. If facial expression duration and intensity influence one’s recognition performance, we hypothesize that self-awareness of facial expressions may also be influenced by facial expression intensity and duration.

We attempt to study self-awareness of facial expressions and the influential factors from two modalities. We further propose a real-time monitoring and video-review paradigm to investigate the awareness rate of a subject based on information from different modalities, specifically, somatosensory feedback information from bodily sensory feedback and visual information from the individual’s facial expression recordings in the video-review condition. In the real-time monitoring condition, participants were instructed to press a key on a keyboard the moment they felt facial movement and then return their facial expression to a neutral state. In the video-review condition, participants were asked to identify any facial movements in their video clips recorded in the real-time condition and press the pause button.

The present study investigated the extent to which people are self-aware of their facial expressions under real-time and video-review conditions and the influencing factors. We hypothesized that awareness of facial expressions would be influenced by both duration and intensity. The present study also sought to calculate the duration and intensity thresholds required for individuals to become aware of their own facial expressions. In addition, we intended to investigate the potential relationship between real-time and video-review awareness.

## Materials and Methods

### Participants

Twenty-seven participants who were naïve to the study’s objectives were recruited (16 females; mean age = 22.59 years, *SD* = 2.17). All participants signed an informed consent form and were told that they had the right to terminate the experimental procedure at any time. The data from four participants were excluded due to technical issues (i.e., camera failed to record because of insufficient memory space). Because we conducted the study during the last month of the academic school year, our data-collection aims were modest – to collect data from at least 25 students and finally collect at least 300 facial expression sample with emotional meanings. The institutional review board (IRB) of the Institute of Psychology, Chinese Academy of Sciences approved the study protocol.

### Apparatus

An Open CV (Open Source Computer Vision Library)-based program was developed to record the time of participants’ key press with relative accuracy and precision during the presentation of each emotional video while simultaneously controlling a high-speed video camera (Logitech Pro C920, recording at 60 fps) that captured participants’ facial activities during each video.

After each emotional video was presented, the program stopped recording and saved the clip containing the participants’ facial activities during the stimulus presentation to the hard drive.

### Materials

Nine videos (5 meant to elicit happiness, 2 meant to elicit disgust, and 2 meant to elicit anger) were selected (see **Table 1**). Seven of the nine videos were chosen from a previous study (Yan et al., 2013), and the other 2 were chosen from the Internet. Twenty additional participants who did not participate in the formal experiment rated the videos by choosing one or two emotion keywords from a list and rated their intensity on a 7-point Likert scale (where 1 denoted not intense and 7 denoted very intense). If words belonging to a certain basic emotion (e.g., happiness) were chosen by one-third of the participants or more, that emotion was considered the main emotion(s) of the video (see **Table 1**) (Yan et al., 2013). We chose happiness, disgust, and anger as the target emotions because facial expressions related to these emotions have been observed to be readily elicited in studies that employed the neutralizing paradigm (Porter and ten Brinke, 2008; Yan et al., 2013). Each video lasted 1–2 min (see **Table 1**). Volume was fixed and controlled across participants.

**Table 1:** Participant ratings of the nine video clips.

Clip no.	Duration (min:sec)	Main emotion	Selection rate	Mean intensity score
1	1:07	Disgust	0.86	4.14
2	1:35	Disgust	0.92	4.33
3	1:57	Anger	0.75	4.5
4	2:24	Anger	0.77	3.92
5	1:18	Happiness	0.92	4
6	1:32	Happiness	0.86	3.07
7	1:16	Happiness	0.86	3.28
8	1:48	Happiness	0.73	3.64
9	1:09	Happiness	0.71	3.17

### Procedure

The participants sat at a table facing a 19-inch, color LCD monitor. A high-speed video camera on a tripod was placed behind the monitor to record the frontal view of the participant’s face.

#### Phase 1: Real-Time Self-monitoring

All participants were tested individually. To obtain “uncontaminated” leaked fast facial expressions that are uncorrupted by various unemotional facial movements (e.g., speaking, blowing the nose and pressing the lips), a facial neutralization paradigm was used (Yan et al., 2013). Participants were motivated to neutralize their faces while they watched high-arousal emotional video clips.

We told the participants that they would view a series of short videos (some of which would be unpleasant) and that we were interested in their ability to control and be aware of their facial movements. The presentation sequence of the experimental videos was counterbalanced across participants. The participants watched the screen closely to maintain a neutral face and were instructed to avoid bodily movement. The participants were also instructed to press the spacebar as soon as they became aware of any facial movement and to return to a neutral face as quickly as possible.

The experimenter monitored the participants’ faces on-line from another monitor. This setup helped the experimenter pre-define certain habitual movements to be verified by the participant after each video was shown. After each video, participants were prompted with a question that required them to rate the emotional valence and intensity of the video.

#### Phase 2: Video Review

The cue-review paradigm proposed by Rosenberg and Ekman (1994) requires participants to watch a replay of a stimulus film and report their emotions at points during the film when they remember having an emotion or an expression on a momentary basis, providing researchers with the opportunity to examine momentary changes in facial expressions of emotion rather than aggregated measures.

After Phase 1 ended, we applied the modified cued-review paradigm by asking participants to review their own facial expressions. Participants were asked to identify any facial movements in these clips and press the pause button. The experimenter recorded the time and asked the participants to recall and verbally report the emotion felt when they displayed the facial expression. These data were used to analyze the awareness rate of the participants’ facial expressions in the video-review setting. Participants received only one chance to decide whether a facial expression had occurred, to match the procedure of Phase 1.

### Coding

Prior to manual coding, all facial movements detected by participants in phase two were classified into two groups based on the participants’ self-reports: (a) facial movements that participants could recall and for which they could report feeling a clear emotion and (b) those verified as habitual movements or facial movements that participants could not recall and for which they could not report feeling a clear emotion. According to the participants’ self-reports, we filtered those frames (via Adobe Premiere 6.0) that were confirmed by the participants to be habitual movements or facial movements that participants could not recall and report feeling a clear emotion. These edited clips were given to two extensively trained coders who coded each frame of the clips for the presence and duration of the expressions on the participants’ faces. The coding procedures employed were similar to a previous study (Yan et al., 2013).

Coding required the coders to code these expressions’ onset times, apex times and offset times by applying the frame-by-frame approach. The apex frame showed the full expression that had a highest intensity for this facial expression. The offset frame was the frame right before a facial movement returned to baseline (Hoffmann et al., 2010). The total duration of these leaked facial expressions was calculated. Coders also rated the intensity of each facial expression from 1 to 7 (where 1 denoted not intense and 7 denoted very intense).

The reliability coefficient of the two coders was calculated according to the equation used in a previous study (Yan et al., 2013). The reliability coefficient of the two coders was 0.82 for all the samples.

## Results

All remaining facial expression samples with durations less than 4 s were included for further analysis.

### Analysis of Real-Time Facial Expression Self-awareness

#### Descriptive Statistics of Real-Time Self-awareness

Of the 353 facial expressions detected by trained coders via the frame-by-frame approach, there were 204 facial expressions in which participants expressed awareness with a keyboard response. This result indicated that the participants’ awareness rate of their facial expressions during the real-time monitoring phase was 57.79% (see **Table 2**). Among the 204 facial expressions for which participants expressed awareness during the real-time condition (hereafter referred to as “real-time aware facial expression”), there were 14 facial expressions that participants reported being unaware of during the video review phase.

**Table 2:** Descriptive statistics for the real-time and post-awareness phases of leaked facial expressions.

		Real-time awareness		Post-awareness	
	*N*	Count	Rate (%)	Count	Rate (%)
All expressions	353	204	57.79	268	75.92

#### The Relationship between Intensity/Duration of Expression and Real-Time Self-awareness

Forty-nine of the 353 expressions had an apex frame but no offset frame; hence, the total duration could not be obtained for these expressions. The remaining 304 expressions were included in the subsequent analysis.

To further explore the relationship between factors such as expression intensity/duration and expression self-awareness in the real-time condition, a logistic regression was employed (McCullagh and Nelder, 1989). The response variable is binary (1/0): aware or unaware under the real-time or video-review condition. The predicting variables are the duration and intensity of the facial expression. **Table 3** shows the logistic regression coefficient estimations.

**Table 3:** The logistic regression parameter estimations.

Awareness condition	Duration and intensity predict awareness	
	Intensity	Duration
Real-time	0.67^∗∗∗^	0.01
Video-review	0.41^∗∗∗^	0.03^∗∗^

*^∗^*p* < 0.05, ^∗∗^*p* < 0.01, ^∗∗∗^*p* < 0.001.*

When both duration and intensity were included in the regression, interesting results arose and suggested that in the real-time condition, the intensity of facial expression is the only significant predictor of facial expression awareness (β = 0.67, *p* < 0.001), whereas duration is not (β = 0.01, *p* > 0.05). The results suggested that facial expression intensity is more important for expression self-awareness than duration is in the real-time condition.

#### Estimated Intensity Threshold in the Real-Time Awareness Condition

Following the logistic regression model fitting, we obtained different estimated equations to explore the variation of awareness on facial expression as a function of facial expression duration and intensity. The estimated logistic regression equation is as follows:

(1) $$\log\left(\frac{p}{1-p}\right)=-2.86+intensity^{*}0.67+duration^{*}0.01$$

**FIGURE 1:**
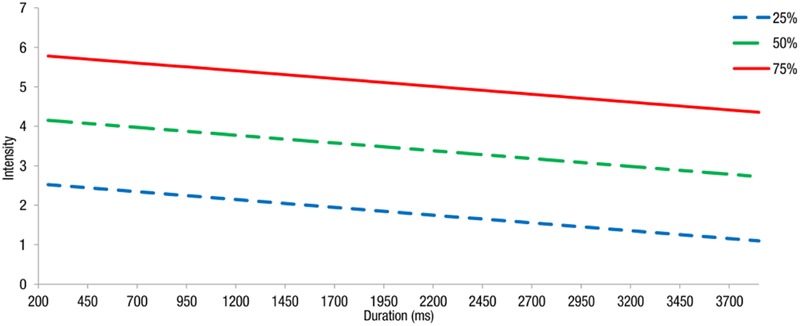
**Estimated intensity thresholds needed by the participants for awareness of self-facial expression with changing duration and different awareness rates (25, 50, and 75%) in the real-time condition.** The expected intensity level for an estimated awareness rate of 95% was not included because it went beyond the maximum intensity setting (i.e., intensity = 7) in this experiment.

Using the regression equations estimated above, we obtained intensity thresholds for different awareness rates (25, 50, 75, and 95%). The logistic regression presents various intensity thresholds needed by the participants for awareness of self-facial expression with changing duration in the real-time condition (**Figure 1**).

### Analysis of Video-Review Facial Expression Self-awareness

#### Descriptive Statistics of Video-Review Facial Expression Self-awareness

Of the 353 facial expressions, there were 268 facial expressions in which participants expressed awareness, estimating the participants’ awareness rate of their facial expressions during the video-review phase as 75.92%. Among the 268 facial expressions in which participants expressed awareness during the video-review phase (hereafter referred to as “video-review aware facial expression”), there were 91 facial expressions that participants reported being unaware of during the real-time monitoring phase.

#### The Relationship between Intensity/Duration of Expression and Video-Review Self-awareness

To investigate the relationship between the intensity/duration of expression and self-awareness in the video-review condition, a similar analysis was conducted on the remaining 304 expressions with a logistic regression (McCullagh and Nelder, 1989). The response variable was binary (1/0): aware or unaware. The predicting variables were the duration and rated intensity of the facial expression.

In contrast to the real-time condition, in the video-review condition, both the intensity (β = 0.41, *p* < 0.001) and the duration (β = 0.03, *p* < 0.01) were significant predictors of facial expression self-awareness. This result suggests that different mechanisms may exist between the real-time and video-review conditions.

#### Estimated Intensity Threshold in the Video-Review Awareness Condition

Following the logistic regression model fitting, similar logistic regression equation was obtained to estimate the probability of awareness on facial expression as a function of facial expression duration and intensity.

(2) $$\log\left(\frac{p}{1-p}\right)=-1.004+intensity^{*}0.41+duration^{*}0.03$$

**FIGURE 2:**
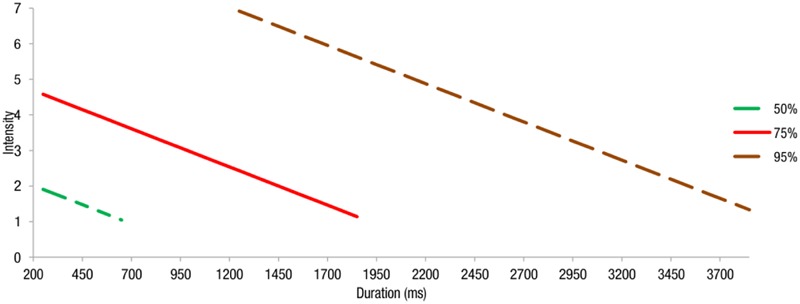
**Estimated intensity thresholds needed by the participants for awareness of self-facial expression with changing duration and different awareness rates (50, 75, and 95%) in the video-review condition.** The expected intensity level for an estimated awareness rate of 25% was not included because it went beyond the minimum intensity setting (i.e., intensity = 1) in this experiment.

Using the regression equations estimated above, we obtained intensity thresholds for different awareness rates (25, 50, 75, and 95%). The logistic regression presents the various intensity thresholds needed by participants for awareness of self-facial expression with changing duration in the video-review condition (**Figure 2**).

### The Relationship between Real-Time and Video-Review Awareness of Facial Expression

Because both real-time and video-review awareness represent the capability of participants to be aware of their own facial expressions, a relationship may exist between these two types of awareness ability. However, no traditional statistical analysis method is available to describe the correlation between these two dichotomous observations, especially because of the inherent bonds of real-time and video-review awareness from the same subject. To address this issue, a novel analysis strategy was proposed. Generalized Estimating Equations (GEE), which was proposed in 1986 and was designed to model repeated measures, has shown great robustness for both the response distribution and various working correlation matrixes (Liang and Zeger, 1986). GEE has been widely and extensively used to model clustered responses, including binary responses (Zhang et al., 2011). Therefore, we applied GEE to investigate the relationship between real-time and video-review awareness. With the observations of each subject taken as a cluster, we fitted real-time awareness as the response variable with a logit link function to investigate to what extent video-review awareness is related to real-time awareness (Zhang et al., 2011).

The results showed a strong relationship between video-review and real-time awareness (*p* < 0.0001), suggesting that video-review awareness is a significant predictor of real-time awareness and that an individual’s ability to be aware of facial expressions in the video-review awareness condition may predict one’s performance in the real-time condition.

## Discussion

### Real-Time Self-awareness of Facial Expressions

The accuracy of self-perception has been a long-standing area of study in psychology (Robins and John, 1997). Many theorists have been less than sanguine about people’s ability to perceive their behavior objectively (Gosling et al., 1998). However, according to previous physiological studies on individuals’ facial expressions (Rinn, 1984; Proske and Gandevia, 2012), some researchers are confident in individuals’ ability to be aware of their facial expressions. Our results reveal that the average real-time awareness rate of all 353 leaked facial expressions was 57.79%. Individuals mainly rely on internally generated somatosensory feedback to form a subjective experience of facial expressions during a real-time phase.

From the perspective of self-perception theory, Laird (1984) suggested that the relation between facial expressions and emotional experience is a particular case of the general relation between behaviors and psychic states. It has been proposed that greater facial expressivity is uniformly associated with greater subjective experience both between and within subjects, indicating that the duration and intensity of facial expressions is associated with subjective experiences of self-produced facial expressions (Adelmann and Zajonc, 1989).

Our results are partly consistent with the above notion, revealing that the duration and intensity of facial expressions are associated with the subjective experience of self-produced facial expressions. To be more specific, in the real-time condition, only the intensity of facial expressions was a significant predictor of self-awareness, whereas duration was not. This result is consistent with Tomkins and Ekman’s statements (Tomkins, 1962; Ekman, 2009). Furthermore, estimated intensity thresholds using logistic regression models show that at certain durations, a higher intensity threshold is needed for a higher awareness rate. This result may be explained by the information the participants employed. In the real-time condition, the participants mainly depended on somatosensory feedback information, such as muscle and skin sensations, to assist their awareness of facial expressions. Other indirect evidence has shown that facial expressions with high intensity are more easily recognized at a higher rate (Herba et al., 2006), which further indicates that intensity, not duration, is the only significant predictor of facial expression self-awareness.

### Video-Review Self-awareness of Facial Expressions

The task in the video-review phase was actually a visual facial change detection task, which was mainly dependent on the visual information from one’s own facial recordings. In the video-review condition, the awareness rate was 75.92%. Various factors have been demonstrated to be related to the accuracy of change detection, such as stimulus duration, interstimulus interval (ISI), intervening masks, and familiarity (Pashler, 1988). Other studies that have employed facial expression recognition have also found that the recognition accuracy rate was related to facial expression intensity (Herba et al., 2006) and duration (Shen et al., 2012).

In the video-review condition, our result was consistent with previous studies and showed that both the intensity and the duration of facial expressions were significant predictors of self-awareness. Furthermore, estimated intensity thresholds using logistic regression models show that at certain durations, a higher intensity threshold is necessitated for a higher awareness rate. Both intensity and duration information are important when the task is to detect changes that occur in participants’ faces in videos.

### The Relationship between Real-Time and Video-Review Awareness of Facial Expressions

Video-review awareness was relatively higher than real-time awareness (75.92% for video-review awareness and 57.79% for real-time awareness). This finding might indicate that awareness based on visual information is more advantageous. Several possibilities might produce the difference between these two conditions. First, in the real-time awareness condition, awareness of facial expressions was spontaneous and instant; the participants were required to monitor their facial expressions and to give their reports immediately when they felt their expression change due to the emotional movie. They possessed immediate somatosensory feedback, but visual information was not available; they could not see their own facial expressions. In the video-review condition, awareness was not spontaneous, and the participants watched their own facial expression videos without a sense of manipulation and muscle action. Second, in the real-time awareness condition, participants needed to watch the elicitation movies while monitoring their facial movements. In the video-review condition, participants only needed to watch their facial recordings and detect any changes in their faces visually. Subjects were more focused in the video-review condition, and the cognitive load was relatively lower. Third, the participants may have had post somatosensory feedback and sensory memory in the video-review condition, which may have facilitated awareness of the same expressions, thus outperforming the real-time condition.

Despite the differences addressed above, both of the awareness conditions represent individuals’ ability to be aware of their own facial expressions. Thus, correlation between these two awareness conditions might exist. Our results suggest a strong relationship between the real-time and video-review awareness of facial expressions. Whether participants were aware or unaware of their facial expressions in the video-review condition significantly predicted their awareness in the real-time condition. As stated in the above passage, after the real-time awareness task, the participants may have had sensory memory of their facial movements. The participants may have used the integrated information from the post somatosensory feedback and visual information in the video-review condition, suggesting that the above two awareness abilities are correlated.

Several limitations should be noted when interpreting our findings. We used a self-report method to study individuals’ self-awareness of their facial expressions. However, self-report measurements may be vulnerable to factors such as self-enhancement or other biases, as previous studies have shown (Gosling et al., 1998). Therefore, researchers should be cautious when interpreting and applying these findings. Furthermore, in the current study, we didn’t include a baseline mood measure prior Phase 1. This measure could be used to better investigate its influence on the outcome in future research. In addition, participants in our study mainly consisted of university students with a limited age range and the number of participants recruited were rather limited. This somewhat limits the generalizability of the present results and it would be interesting to investigate participants from an older age group in future research, as elderly participants are generally associated with less negative face-emotion processing and physiological changes to emotion are also different to student cohort.

In the present study, considering the huge individual difference in the number of leaked facial expression and thus the awareness rate, we didn’t analyze the data from the individual level, which might be one limitation. In the next study, we consider to separate the participants into different groups according to their facial expression expressivity, with subjects show relatively more numbers of facial expression in daily life into high expressivity group and less numbers of facial expression in daily life (e.g., people with poker face) into low expressivity group.

## Author Contributions

FQ and XF designed the experiment and wrote the manuscript. FQ, W-JY, Y-HC, and HZ performed the experiment and analyzed the collecting data. FQ, KL, Y-HC, and XF revised the manuscript.

## Conflict of Interest Statement

The authors declare that the research was conducted in the absence of any commercial or financial relationships that could be construed as a potential conflict of interest.

## References

[B1] Adelmann P. K. Zajonc R. B. (1989). Facial efference and the experience of emotion. *Annu. Rev. Psychol.* 40 249–280. 10.1146/annurev.ps.40.020189.001341 2648977

[B2] Ansfield M. E. DePaulo B. M. Bell K. L. (1995). Familiarity effects in nonverbal understanding: recognizing our own facial expressions and our friends’. *J. Nonverbal Behav.* 19 135–149. 10.1007/BF02175501

[B3] Barr C. L. Kleck R. E. (1995). Self-other perception of the intensity of facial expressions of emotion: do we know what we show? *J. Pers. Soc. Psychol.* 68 608-618 10.1037/0022-3514.68.4.608 7738767

[B4] Barrett L. F. Mesquita B. Ochsner K. N. Gross J. J. (2007). The experience of emotion. *Annu. Rev. Psychol.* 58 373-403 10.1146/annurev.psych.58.110405.085709 PMC1934613 17002554

[B5] De Vignemont F. (2014). A multimodal conception of bodily awareness. *Mind* 123 989–1020. 10.1093/mind/fzu089

[B6] DePaulo B. M. (1992). Nonverbal behavior and self-presentation. *Psychol. Bull.* 111 203-243 10.1037/0033-2909.111.2.203 1557474

[B7] Ekman P. (2009). “Lie catching and microexpressions,” in *The Philosophy of Deception* ed. Martin C. (Oxford: Oxford University Press) 118–133. 10.1093/acprof:oso/9780195327939.003.0008

[B8] Ekman P. Friesen W. V. (1971). Constants across cultures in the face and emotion. *J. Pers. Soc. Psychol.* 17 124-129 10.1037/h0030377 5542557

[B9] Ekman P. Freisen W. V. Ancoli S. (1980). Facial signs of emotional experience. *J. Pers. Soc. Psychol.* 39 1125-1134 10.1037/h0077722

[B10] Gosling S. D. John O. P. Craik K. H. Robins R. W. (1998). Do people know how they behave? Self-reported act frequencies compared with on-line codings by observers. *J. Pers. Soc. Psychol.* 74 1337-1349 10.1037/0022-3514.74.5.1337 9599447

[B11] Herba C. M. Landau S. Russell T. Ecker C. Phillips M. L. (2006). The development of emotion-processing in children: effects of age, emotion, and intensity. *J. Child Psychol. Psychiatry* 47 1098–1106. 10.1111/j.1469-7610.2006.01652.x 17076748

[B12] Hess U. Senecal S. Thibault P. (2004). Do we know what we show? Individuals’ perceptions of their own emotional reactions. *Curr. Psychol. Cogn.* 22 247–266.

[B13] Heuer K. Lange W. G. Isaac L. Rinck M. Becker E. S. (2010). Morphed emotional faces: emotion detection and misinterpretation in social anxiety. *J. Behav. Ther. Exp. Psychiatry* 41 418–425. 10.1016/j.jbtep.2010.04.005 20511123

[B14] Hochschild A. R. (1979). Emotion work, feeling rules, and social structure. *Am. J. Sociol.* 85 551–575. 10.1086/227049

[B15] Hoffmann H. Traue H. C. Bachmayr F. Kessler H. (2010). Perceived realism of dynamic facial expressions of emotion: optimal durations for the presentation of emotional onsets and offsets. *Cogn. Emot.* 24 1369–1376. 10.1080/02699930903417855

[B16] Laird J. D. (1984). The real role of facial response in the experience of emotion: a reply to Tourangeau and Ellsworth, and others. *J. Pers. Soc. Psychol.* 47 909–917. 10.1037/0022-3514.47.4.909 501520

[B17] Liang K.-Y. Zeger S. L. (1986). Longitudinal data analysis using generalized linear models. *Biometrika* 73 13–22. 10.1093/biomet/73.1.13

[B18] McCullagh P. Nelder J. A. (1989). *Generalized Linear Models* Vol. 37 Boca Raton, FL: CRC press 10.1007/978-1-4899-3242-6

[B19] North M. S. Todorov A. Osherson D. N. (2010). Inferring the preferences of others from spontaneous, low-emotional facial expressions. *J. Exp. Soc. Psychol.* 46 1109–1113. 10.1016/j.jesp.2010.05.021

[B20] North M. S. Todorov A. Osherson D. N. (2012). Accuracy of inferring self- and other-preferences from spontaneous facial expressions. *J. Nonverbal Behav.* 36 227–233. 10.1007/s10919-012-0137-6

[B21] Pashler H. (1988). Familiarity and visual change detection. *Percept. Psychophys.* 44 369–378. 10.3758/BF03210419 3226885

[B22] Porter S. ten Brinke L. (2008). Reading between the lies: identifying concealed and falsified emotions in universal facial expressions. *Psychol. Sci.* 19 508–514. 10.1111/j.1467-9280.2008.02116.x 18466413

[B23] Proske U. Gandevia S. C. (2012). The proprioceptive senses: their roles in signaling body shape, body position and movement, and muscle force. *Physiol. Rev.* 92 1651–1697. 10.1152/physrev.00048.2011 23073629

[B24] Riggio R. E. Widaman K. F. Friedman H. S. (1985). Actual and perceived emotional sending and personality correlates. *J. Nonverbal Behav.* 9 69–83. 10.1007/BF00987139

[B25] Rinn W. E. (1984). The neuropsychology of facial expression: a review of the neurological and psychological mechanisms for producing facial expressions. *Psychol. Bull.* 95 52-77 10.1037/0033-2909.95.1.52 6242437

[B26] Robins R. W. John O. P. (1997). Effects of visual perspective and narcissism on self-perception: is seeing believing? *Psychol. Sci.* 8 37–42. 10.1111/j.1467-9280.1997.tb00541.x

[B27] Rosenberg E. L. Ekman P. (1994). Coherence between expressive and experiential systems in emotion. *Cogn. Emot.* 8 201–229. 10.1016/j.biopsycho.2013.09.003

[B28] Shen X.-B. Wu Q. Fu X.-L. (2012). Effects of the duration of expressions on the recognition of microexpressions. *J. Zhejiang Univ. Sci. B* 13 221–230. 10.1631/jzus.B1100063 22374615 PMC3296074

[B29] Tomkins S. S. (1962). *Affect, Imagery, Consciousness: The Positive Affects* Vol. I New York City, NY: Springer Publishing Company.

[B30] Yan W.-J. Wu Q. Liang J. Chen Y.-H. Fu X. (2013). How fast are the leaked facial expressions: the duration of micro-expressions. *J. Nonverbal Behav.* 37 217–230. 10.1007/s10919-013-0159-8

[B31] Zhang H. Xia Y. Chen R. Gunzler D. Tang W. Tu X. (2011). Modeling longitudinal binomial responses: implications from two dueling paradigms. *J. Appl. Stat.* 38 2373–2390. 10.1080/02664763.2010.550038

